# The Late Meetings in Baltimore

**Published:** 1895-06

**Authors:** 


### EDITORIAL.

The office of THE JOURNAL is in room 330 Equitable Building.

The editors are not responsible for views expressed by contributors.

Articles for publication should reach this office not later than the 15th instant.

Address all communications, and make all remittances payable to THE ATLANTA MEDICAL AND SURGICAL JOURNAL, Atlanta, Ga.

Reprints of articles will be furnished, when desired, at a nominal cost. Requests for the same should always be made *on the manuscript*.

### THE LATE MEETINGS IN BALTIMORE.

The city of Baltimore has attracted a great concourse of medical men recently, commencing with the Medical and Chirurgical Faculty of the State of Maryland, from April 23d to 26th inclusive. The next in order coming the American Academy of Medicine on the 4th and 6th of May, and finally the American Medical Association from May 7th to 10th inclusive. In connection with these were meetings of the American Medical College Association, the American Medical Editors' Association, and the American Medical Publishers' Association.

The medical men and citizens of Baltimore rendered the stay of the visitors pleasant by public and private receptions and entertainments, so that all came away much pleased with the attentions received, and congratulated themselves upon being brought into social relations not only with their colleagues, but with the ladies and gentlemen of this progressive and hospitable city.

This was the ninety-seventh annual session of the Medical and Chirurgical Society, the President being Dr. Robert W. Johnson. His opening address was upon "Pernicious Delay in Surgical Cases," which was ably treated by him. "Typhoid Fever in Country Districts" was the subject for general discussion before the Society, in which Dr. William Osler led. A report of several cases of "Bullet Wounds of the Liver and Stomach" was presented by Dr. L. McLane Tiffany. "The Causation of Nervous Diseases" was the title of the annual oration by Dr. M. Allen Starr of New York. This address was much applauded, and requested for publication.

The American Academy of Medicine, under the Presidency of Dr. J. McFadden Gaston, of this city, offered a class of papers of great interest, not only to the profession, but to all who are engaged in the consideration of medical sociology; and the scope of investigation in this department is opening a wide field of usefulness for the Academy in the future. It is doubtless generally understood that the fellowship of this organization consists of those graduates of medicine whose literary course gives the degree of A.B. or its equivalent standing; and the address of the president, urged not only a liberal preliminary education, but preparatory instruction in the office of a preceptor as requisite for entering upon a course of lectures in a medical college. It was claimed, however, in the president's address, that many of the medical colleges which have adopted the three years' graded course are not prepared as yet to enter upon the four years' graded course insisted upon by the American Medical College Association. To quote his own words: "While the endowed institutions are prepared to assume the responsibilities of such a step, and it is commendable in independent organizations to afford the very best opportunities for the highest attainments of those who can devote this additional time to preparatory training for the medical profession, a large majority of the medical colleges cannot now undertake more than a three years' graded course. They are ready to make a decided advance by the adoption of the three years' graded course; and many of them have already entered upon the curriculum of instruction on this basis. This is so marked an improvement upon the old two years' course, that we should congratulate the profession upon this evidence of progress, and be content to await further developments to authorize an increase of another year."

We note an editorial in the *Medical Record* (May 18) agreeing in the main with the above. There seeems to have developed in late years a sort of "four-year-course fanaticism among some of those who are in the propaganda for higher education." We quite agree with the writer, that with the preliminary education now required, and a certain amount of hospital work after graduation, there is no necessity for colleges falling over one another in rushing their course of lectures and recitations up to four years.

However, the question of a four years' graded course was finally disposed of by the American Medical College Association, at its meeting on Wednesday afternoon, May 8th, deciding by a large majority in favor of entering upon this course at the opening sessions of the medical colleges in the fall of the current year.

The meeting of the American Medical Association was largely attended, and the proceedings in the general sessions and in the sections were of unusual interest; but our space does not admit of more than a passing reference to them at present.

The annual address of the President, Dr. McLean, was upon "A Few Living Issues in the Practice of Medicine, and What Came of Them." He alluded briefly to certain landmarks in the history of medicine, and entered a strong plea for more systematic and improved plans of medical education. He also advocated a national bureau of health, with a competent medical officer in the cabinet. It was voted that this part of the address be referred with recommendation to the proper officials at Washington.

A matter which has been causing considerable agitation, in regard to the management of the *Journal* touching advertisements, was satisfactorily settled by passing a resolution requiring that the full formulas of medicinal compounds shall accompany their announcement in the paper, and that all not conforming to this rule shall be excluded. The cheers which greeted this action indicated its hearty approval by a very decided majority of the Association; and though it may lessen materially the income from this class of advertisements, it places the *Journal of the American Medical Association* upon a high plane which ought to secure an increase of the number of subscribers, in compensation for the shortcoming from such advertisements in its pages. We congratulate the Association for taking this position.

More than the usual amount of work was done in the different Sections of the Association, embracing those of the Practice of Medicine, Surgery and Anatomy, Obstetrics and Gynecology, Eye, Ear and Throat, State Medicine, etc. The address on General Medicine was delivered by Dr. William E. Quine, of Chicago, entitled "The Malarial Disorders of Large Cities." Dr. C. A. Wheaton, of St. Paul, delivered the address on Surgery, in which he advocated greater conservatism. The address on State Medicine was delivered by Dr. H. D. Holton, of Vermont. He emphasized the importance of State Boards of Health, and advocated a national department of public health and a uniform system of quarantine.

It was the general verdict that this was one of the most successful meetings in the history of the Association.

The next meeting will be held in Atlanta the first Tuesday in May, 1896.

The officers of the American Medical Association for the coming year are as follows:

President, Dr. R. Beverly Cole, of San Francisco.

Vice-Presidents, Dr. Julian J. Chisholm, of Baltimore; Dr. John C. Legrand, of Anniston, Ala.; Dr. Augustus B. Clark, of Cambridge, Mass.; Dr. T. P. Satterwhite, of Louisville, Ky.

Treasurer, Dr. Henry P. Newman, of Chicago, Ill.

Secretary, Dr. W. B. Atkinson, of Philadelphia, Pa.

Assistant Secretary, Dr. J. McFadden Gaston, Jr., of Atlanta.

Editor of the *Journal of the Association*, Dr. John B. Hamilton, of Chicago.

Trustees, Dr. Alonzo Garcelon, of Maine; Dr. I. N. Love, of Missouri; Dr. James E. Reeves, of Tennessee.

Judicial Committee, Dr. N. S. Davis, of Chicago, Ill.; Dr. H. D. Didama, of New York; Dr. John Morris, of Maryland; Dr. W. E. B. Davis, of Alabama; Dr. George W. Broome, of Missouri; Dr. D. W. Smouse, of Iowa; Dr. M. B. Ward, of Kansas; Dr. W. M. Welch, of Pennsylvania.

Annual addresses by the following: On Surgery, Dr. Nicholas Senn, of Chicago; on Medicine, Dr. William Osler, of Baltimore; on State Medicine, Dr. G. H. Rohe, of Baltimore.

Chairman Committee of Arrangements at Atlanta, Dr. W. F. Westmoreland, Atlanta, Ga.

